# Concurrent Presence of Obstructive Sleep Apnea and Elevated Homocysteine Levels Exacerbate the Development of Hypertension: A KoGES Six-year Follow-up Study

**DOI:** 10.1038/s41598-018-21033-5

**Published:** 2018-02-08

**Authors:** Jinkwan Kim, Seung Ku Lee, Dae Wui Yoon, Chol Shin

**Affiliations:** 1 Department of Biomedical Laboratory Science, College of Health Science, Jungwon University, Geo-San, Republic of Korea; 2 Department of Pulmonary Sleep and Critical Care Medicine Disorder Center, College of Medicine, Korea University, Ansan, Republic of Korea; 3 Institute of Human Genomic Study, Korea University Ansan Hospital, Korea University, Ansan, Republic of Korea

## Abstract

Accumulating evidence has revealed that obstructive sleep apnea (OSA) and high homocysteine (Hcy) levels play important roles in the increased risk of hypertension and cardiovascular disease. We investigated whether the presence of elevated Hcy levels among individuals with OSA increase the risk of hypertension in a cohort study. A total of 1825 participants were selected from the cohort study. A high homocysteine level (Hcy) was defined as those in the 75th percentile of Hcy levels of the study cohort. The prevalence of hypertension was higher among subjects with OSA and high Hcy levels than among the other groups stratified by the presence of OSA and high Hcy levels. The incidence of hypertension at 6-year follow-up was: Hcy[−]/OSA[−] vs. Hcy[+]/OSA[−] vs. Hcy[−]/OSA[+] vs. Hcy[+]/OSA[+], 14.2% vs. 19.8% vs. 24.2% vs. 36.1%. After adjusting for confounding factors, subjects with OSA and high Hcy levels had a 1.86-fold risk of developing hypertension compared to those without OSA and high Hcy levels. Moderate to severe OSA group with the highest tertile of Hcy levels had a 2.31-fold increased risk of developing hypertension. Interaction between Hcy and OSA on development of hypertension was significant, suggesting that these conditions may constitute an important determinant.

## Introduction

Obstructive sleep apnea (OSA) is characterized by repeated events of partial or complete upper airway obstruction during sleep, leading to hypoxemia and sleep fragmentation. Increasing evidence from several lines of investigation strongly supports that OSA is independently associated with increased risk for hypertension and cardiovascular disease (CVD)^1–3^. Though the underlying mechanisms by which OSA leads to increased risk of CVD are not clearly evident, increased sympathetic activity, activation and propagation of oxidative stress by intermittent hypoxia and reoxygenation, and systemic inflammation are mechanistically involved in the pathogenesis of CVD in OSA^4,5^. Substantial data from large cross-sectional studies demonstrated that OSA is independently associated with increased risk of hypertension^2,3,6^, and prospective studies have produced conflicting results on the causal relationship between incidence of hypertension and OSA, owing to methodological limitations^6–9^.

Homocysteine (Hcy) is an intermediate product of metabolism of an essential sulfur-containing amino acid in the methionine to cysteine pathway^10^. It has been suggested that an elevated Hcy level is associated with an increased risk for CVD and is pathophysiologically involved in the promotion of cardiovascular events by inducing endothelial dysfunction and coagulation abnormalities^10,11^. Even though there are conflicting results between studies addressing Hcy levels in OSA^12^, several studies in the clinical setting reported that increased levels of Hcy were observed in adults with OSA^13–15^. Recently, results from a meta-analysis also showed a significant association between OSA and plasma Hcy, suggesting that elevated Hcy might be one of the mechanisms responsible for OSA-related cardiovascular complications^10^. However, no study has been published regarding whether the co-existence of an elevated Hcy level and OSA increased the risk of hypertension and exacerbated the development of hypertension in a large prospective study. Therefore, we hypothesized that the presence of elevated Hcy levels among individuals with OSA would accelerate the risk of hypertension in a cohort study.

## Results

### Study Population

We performed a prospective cohort study of 1825 participants derived from the Korean Genome and Epidemiology Study initiated in 2001. Participants underwent baseline PSG and continued to participate for an average of 5.89 years. Of the 1825 participants at baseline examination, 210 subjects (114 males and 96 females) dropped out over the 6-year follow-up period (non-response rate, 11.5%). There were no significant differences between the respondents and the non-respondents in regard to gender, smoking status, alcohol use, BMI, or AHI data from the PSG (p > 0.05). However, the mean age was significantly higher in the non-respondents than the respondents (non-respondents vs. respondents, 57.5 ± 8.5 vs. 55.2 ± 7.0 years old, p < 0.05). At baseline, the participant’s mean age was 55.5 years old, and 52.8% was male. The general characteristics of the participants for the present study are presented in Table 1. The participants were divided into two groups—OSA and non-OSA—and these two groups were further divided into two groups based on Hcy levels—low and high. Among the non-OSA participants, the average Hcy for those in the “low” and “high” Hcy groups at baseline were 9.9 and 16.1 μM; among the OSA participants, the average Hcy for those in the “low” and “high” Hcy groups were 10.3 and 16.1 μM. At a 2-year follow-up, these values increased to 12.2, 17.0, 12.8, and 16.9 μM, respectively. PSG data, including AHI and oxygen saturation (SaO_2_) nadir, also showed a significant group difference (p < 0.01). Systolic and diastolic BP, glucose, triglycerides, and HDL cholesterol levels were significantly different among the four groups (p < 0.01); however, total cholesterol was similar. Moreover, a significant difference for vitamin supplement intake was found in participants with low vs. high Hcy among non-OSA and OSA groups (p < 0.01).

**Table 1:** General characteristics of study participants according to the presence of high homocysteine level and OSA^1)^.

	Non-OSA		OSA		p- value^2^
	Hcy (−)	Hcy (+)	Hcy (−)	Hcy (+)	
Sample size, n (%)	761 (41.7)	208 (11.4)	606 (33.2)	250 (13.7)	—
Age (years)	53.2 ± 6.1	55.7 ± 8.1^&^	56.9 ± 7.2^‡^	58.6 ± 7.7^†^	<0.01
BMI (kg/m^2^)	23.7 ± 2.6	24.2 ± 2.5^§^	25.4 ± 2.9^‡^	25.3 ± 2.6^†^	<0.01
ΔBMI (kg/m^2^)^*^	0.04 ± 1.18	−0.10 ± 1.3	0.03 ± 1.3	−0.15 ± 1.2	0.115
ESS	5.9 ± 4.5	5.4 ± 3.8	5.8 ± 3.9	5.5 ± 5.5	0.375
Male, n (%)	522 (49.5)	152 (47.8)	203 (64.2)	91 (62.3)	<0.001
Current smoker, n (%)	85 (11.2)	48 (23.1)	65 (10.7)	62 (24.8)	<0.01
Current drinker, n (%)	327 (43.0)	130 (62.5)	306 (50.5)	167 (66.8)	<0.01
Medication for HTN, n (%)	171 (16.2)	74 (23.3)	111 (35.1)	57 (39.0)	<0.01
Medication for DM, n (%)	92 (9.7)	44 (16.2)	50 (17.9)	32 (25.4)	<0.01
Vitamin supplement intake, n (%)	226 (29.7)	39 (18.8)	165 (27.2)	36 (14.4)	<0.01
Systolic BP at baseline (mmHg)	108.3 ± 13.4	114.2 ± 14.3^§^	113.2 ± 13.9^‡^	117.2 ± 12.9^†^	<0.01
Diastolic BP at baseline (mmHg)	72.4 ± 9.2	76.8 ± 9.4^§^	75.3 ± 9.4^‡^	78.0 ± 9.1^†^	<0.01
Systolic BP at follow-up (mmHg)	112.5 ± 13.5	116.9 ± 14.7^§^	117.0 ± 13.2^‡^	119.2 ± 13.4^†^	<0.01
Diastolic BP at follow-up (mmHg)	72.8 ± 9.1	75.4 ± 9.4^§^	74.6 ± 9.1^‡^	75.8 ± 10.4^†^	<0.01
AHI (events/hour)	1.8 ± 1.4	2.2 ± 1.4	13.6 ± 9.6^‡^	13.0 ± 9.4^†^	<0.01
(Median, IQR)	(2.90, 1.1–5.4)	(3.05, 1.1–5.8)	(15.5, 11.9–21.6)	(16.0, 12.3–24.4)	
SaO_2_ nadir (%)	90.3 ± 2.7	89.8 ± 3.3	84.1 ± 4.6^‡^	84.6 ± 4.9^†^	<0.01
(Median, IQR)	(90.0, 87.0–92.0)	(90.0, 87.0–91.0)	(84.0, 80.0–86.0)	(83.0, 79.0–86.0)	
Fasting glucose (mg/dl)	95.7 ± 29.4	103.0 ± 25.0	103.1 ± 34.1^‡^	103.9 ± 36.8^†^	<0.01
Total cholesterol (mg/dl)	199.5 ± 34.5	200.7 ± 35.2	201.0 ± 34.7	199.9 ± 35.5	0.877
HDL cholesterol (mg/dl)	46.2 ± 10.9	43.8 ± 10.7^&^	44.3 ± 10.4^‡^	42.4 ± 9.7^†^	<0.01
Triglycerides (mg/dl)	121.6 ± 72.8	146.3 ± 88.3^&^	148.1 ± 95.8^‡^	160.2 ± 92.3^†^	<0.01
Hcy at baseline (µmol/L) (log transformed)	9.9 ± 1.8	16.1 ± 2.9^&^	10.3 ± 1.7^‡^	16.1 ± 2.6^†^	<0.01
	(0.98 ± 0.08)	(1.20 ± 0.06)	(1.00 ± 0.07)	(1.20 ± 0.06)	
Hcy at follow-up (µmol/L)^* *^ (log transformed)	12.0 ± 2.5	17.0 ± 5.1^&^	12.8 ± 3.5^‡^	16.9 ± 5.1^†^	<0.01
	(1.07 ± 0.08)	(1.21 ± 0.11)	(1.09 ± 0.09)	(1.21 ± 0.10)	

Abbreviations: BMI, body mass index; ESS, Epworth sleepiness scale; HTN, hypertension; DM, diabetes; BP, blood pressure; AHI, apnea hypopnea index; IQR, interquartile range; HDL, high-density lipoprotein; Hcy, homocysteine.

^1)^Scale variables are summarized with mean ± SD; Statistical significance was estimated after log transformation.

^2)^The combinatory association is significant for a p-value < 0.05.

*A total of 1615 subjects were included in the analysis.

**Data from 1763 subjects at a 2-year follow-up were included in the analysis.

^†^p < 0.05, OSA with high Hcy vs. non-OSA with low Hcy

^‡^p < 0.05, OSA with low Hcy vs. non-OSA with low Hcy

^§^p < 0.05, Non-OSA with high Hcy vs. non-OSA with low Hcy

^&^p < 0.05, Non-OSA with high Hcy vs. non-OSA with high Hcy.

### The Association between the Risk of Hypertension, Severity of OSA, and High Hcy Level

The prevalence and incidence of hypertension according to severity of OSA at the 6-year follow-up are shown in Table 2. The prevalence of hypertension was the highest among the participants with moderate to severe OSA. Similarly, the incidence of hypertension at the 6-year follow-up period increased in a dose-dependent manner according to the severity of OSA (non-OSA vs. mild OSA vs. moderate to severe OSA, 15.2% vs. 25.2% vs. 32.8%, respectively, p < 0.001). A multivariate analysis adjusted for age, gender, smoking, alcohol status, BMI, DM medication, fasting glucose, HDL cholesterol, and triglycerides at baseline as well as ΔBMI at the 6-year follow-up showed that participants with moderate to severe OSA (AHI ≥ 15) had a 1.74-fold (95% CI, 1.10–2.72, p < 0.05) increased risk for developing hypertension compared to those without OSA (AHI < 5). Moreover, after adjustment for age, gender, smoking, alcohol status, BMI, DM medication, vitamin supplement intake, fasting glucose, HDL cholesterol, and triglycerides in a multivariate analysis, we found that participants with high Hcy levels had a 2.01-fold increased risk of hypertension (OR, 2.01, 95% CI, 1.59–2.52), comparted to those without high Hcy level (data not shown). However, after adjustment with age, gender, smoking, alcohol status, BMI, DM medication, fasting glucose, HDL cholesterol, and triglycerides at baseline as well as ΔBMI at the 6-year follow-up, the odds ratio for the developing hypertension in participants with a high Hcy level as compared to those with low Hcy levels was not significant (OR, 1.17, 95% CI, 0.84–1.61; p = 0.34).

**Table 2:** Estimated odds ratios for the risk of hypertension at baseline and the development of hypertension at a 6-year follow-up according to severity of OSA.

	Severity of OSA			p-value ^1)^
	AHI < 5	5 ≤ AHI < 15	AH I≥ 15	
	***Odds ratios for the risk of hypertension (95% CI)***			
Sample size, n (%)	969 (53.1)	607 (33.3)	249 (13.6)	—
**Prevalence, n (%)**	**191 (19.7)**	**201 (33.1)**	**114 (45.8)**	**<0.001**
Unadjusted	Reference	2.01 (1.60–2.54)^&^	3.44 (2.56–4.62)^&^	<0.001
Adjusted, Model 1	Reference	1.32 (1.03–1.70)^§^	1.89 (1.36–2.61)^&^	<0.001
	***Odds ratios for the development of hypertension (95% CI)***			
Sample size, n (%)^2)^	706 (59.3)	357 (30.0)	128 (10.7)	—
**Incidence, n (%)**	**107 (15.2)**	**90 (25.2)**	**42 (32.8)**	**<0.001**
Unadjusted	Reference	1.88 (1.37–2.58)^&^	2.73 (1.79–4.17)^&^	<0.001
Adjusted, Model 2	Reference	1.31 (0.94–1.85)	1.74 (1.10–2.72)^§^	<0.01

^&^P < 0.01, ^§^P < 0.05.

^1)^The combinatory association is significant for a p-value < 0.05.

^2)^A total of 1191 participants out of 1825, who did not have hypertension at baseline, were included in the analysis.

Model 1: Adjusted for age, gender, smoking, alcohol status, BMI, DM medication, fasting glucose, HDL cholesterol, and triglycerides

Model 2: Adjusted for age, gender, smoking, alcohol status, BMI, DM medication, fasting glucose, HDL cholesterol, and triglycerides at baseline as well as BMI change (ΔBMI) at the 6-year follow-up.

### Odd Ratios for the Risk of Hypertension According to the Concurrent Presence of High Hcy Levels and OSA

The prevalence and incidence of hypertension among the four groups stratified by the presence of high Hcy and OSA are shown in Table 3. The prevalence of hypertension was significantly different (Hcy[−]/OSA[−] vs. Hcy[+]/OSA[−] vs. Hcy[−]/OSA[+] vs. Hcy[+]/OSA[+], 16.3% vs. 32.2% vs. 32.7% vs. 46.8%, respectively, p < 0.001). Moreover, there was also a significant difference in the incidence of hypertension at the 6-year follow-up among the four groups (Hcy[−]/OSA[−] vs. Hcy[+]/OSA[−] vs. Hcy[−]/OSA[+] vs. Hcy[+]/OSA[+], 14.2% vs. 19.8% vs. 24.2% vs. 36.1%, p < 0.001). In order to estimate the odds ratios for the likelihood of hypertension according to the presence of high Hcy and OSA, univariate and multiple logistic regression analyses were performed. The univariate analysis showed the odds ratios for hypertension among OSA participants with low and high Hcy levels were 2.49 (95% CI, 1.92–3.22; p < 0.001) and 4.51 (95% CI, 3.30–6.18; p < 0.001), respectively compared to the non-OSA participants with low Hcy levels. The multivariate analysis with adjustment for age, gender, smoking, alcohol status, BMI, DM medication, vitamin supplement intake, fasting glucose, HDL cholesterol, and triglycerides showed OSA participants with high Hcy levels had a 2.71-fold increase (95% CI, 1.97–4.05; p < 0.05) in the risk of hypertension compared to non-OSA participants with low Hcy levels. Moreover, Table 3 shows adjusted odds ratios estimated for the risk of developing hypertension at the 6-year follow-up among the four groups of participants who did not have hypertension at baseline. After adjustment for age, gender, smoking, alcohol status, BMI, DM medication, vitamin supplement intake, fasting glucose, HDL cholesterol, and triglycerides at baseline as well as ΔBMI at the 6-year follow-up in a regression model, OSA participants with a high Hcy level had a significantly higher risk of developing hypertension at the follow-up (OR, 1.82, 95% CI, 1.15–3.09; p < 0.05), as compared to non-OSA participants having low Hcy levels.

**Table 3:** Estimated odds ratios for the risk of hypertension at baseline and the development of hypertension during the 6-year follow-up according to the combinatory presence and absence of high homocysteine levels and OSA.

	Hcy (−)/	Hcy (+)	Hcy (−)/	Hcy (+)/	p-value ^1)^
	OSA (−)	/OSA (−)	OSA (+)	OSA (+)	
	***Odds ratios for the risk of hypertension (95% CI)***				
Sample size, n (%)	761 (41.7)	208 (11.4)	606 (33.2)	250 (13.7)	—
**Prevalence, n (%)**	**124 (16.3)**	**67 (32.2)**	**198 (32.7)**	**117 (46.8)**	**<0.001**
Unadjusted	Reference	2.44 (1.72–3.45)^&^	2.49 (1.92–3.22)^&^	4.51 (3.30–6.18)^&^	<0.001
Adjusted, Model 1	Reference	2.00 (1.38–2.93)^&^	1.54 (1.17–2.06)^&^	2.71 (1.97–4.05)^&^	<0.001
	***Odds ratios for the development of hypertension (95% CI)***				
Sample size, n (%)^2)^	580 (48.7)	126 (10.6)	363 (30.5)	122 (10.2)	—
**Incidence, n (%)**	**82 (14.2)**	**25 (19.8)**	**88 (24.2)**	**44 (36.1)**	**<0.001**
Unadjusted	Reference	1.50 (0.91–2.46)	1.94 (1.39–2.71)^&^	3.42 (2.20–5.30)^&^	<0.001
Adjusted, Model 2	Reference	1.10 (0.65–1.87)	1.36 (0.95–1.94)	1.82 (1.15–3.09)^§^	<0.001

^&^P < 0.01, ^§^P < 0.05.

^1)^The combinatory association is significant for a p-value < 0.05.

^2)^A total of 1191 participants out of 1825, who did not have hypertension at baseline, were included in the analysis.

Model 1: Adjusted for age, gender, smoking, alcohol status, BMI, DM medication, vitamin supplement intake, fasting glucose, HDL cholesterol, and triglycerides

Model 2: Adjusted for Adjusted for age, gender, smoking, alcohol status, BMI, DM medication, vitamin supplement intake, fasting glucose, HDL cholesterol, and triglycerides at baseline as well as BMI change (ΔBMI) at the 6-year follow-up.

### Odd Ratios for the Risk of Hypertension According to the Coexistence of High Homocysteine Levels and Moderate to Severe OSA

**Figure 1:**
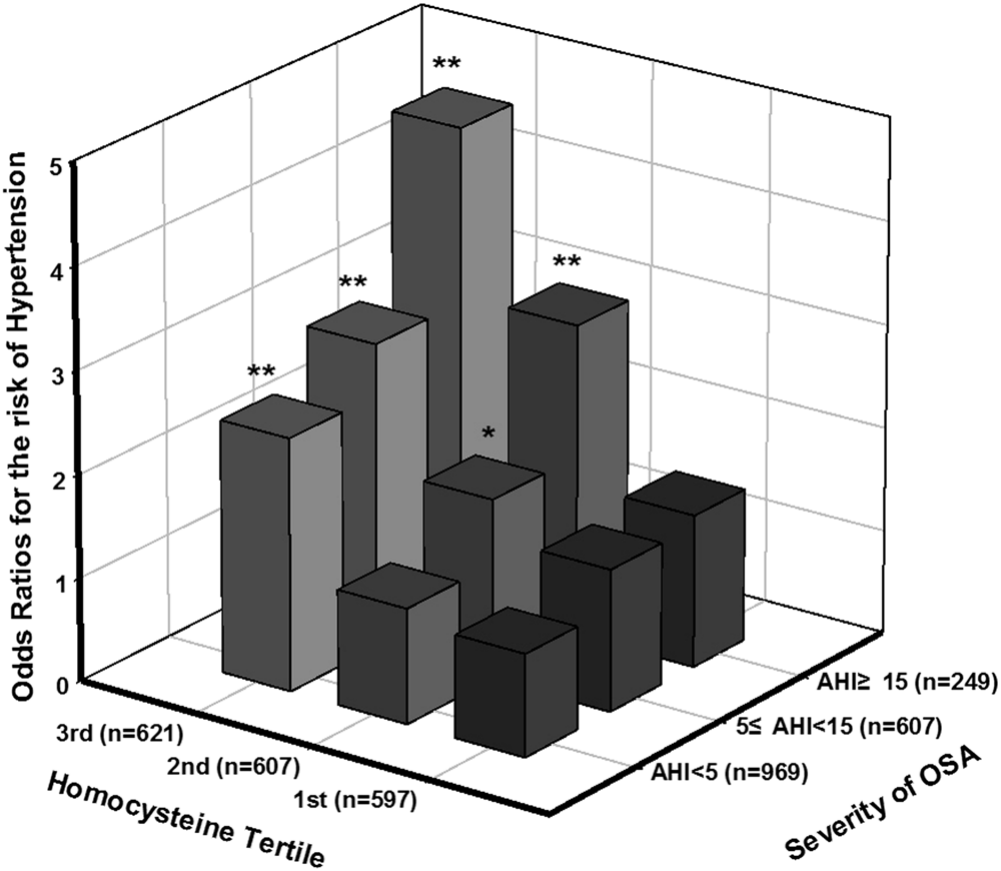
Estimated odds ratios for the risk of hypertension according to combined tertile homocysteine levels and OSA severity. The odd ratios were estimated after adjustment for age, gender, smoking, alcohol status, BMI, DM medication, vitamin supplement intake, fasting glucose, HDL cholesterol, and triglycerides at baseline (n = 1825). P-value for interaction = 0.604. *p < 0.05, **p < 0.01.

**Figure 2:**
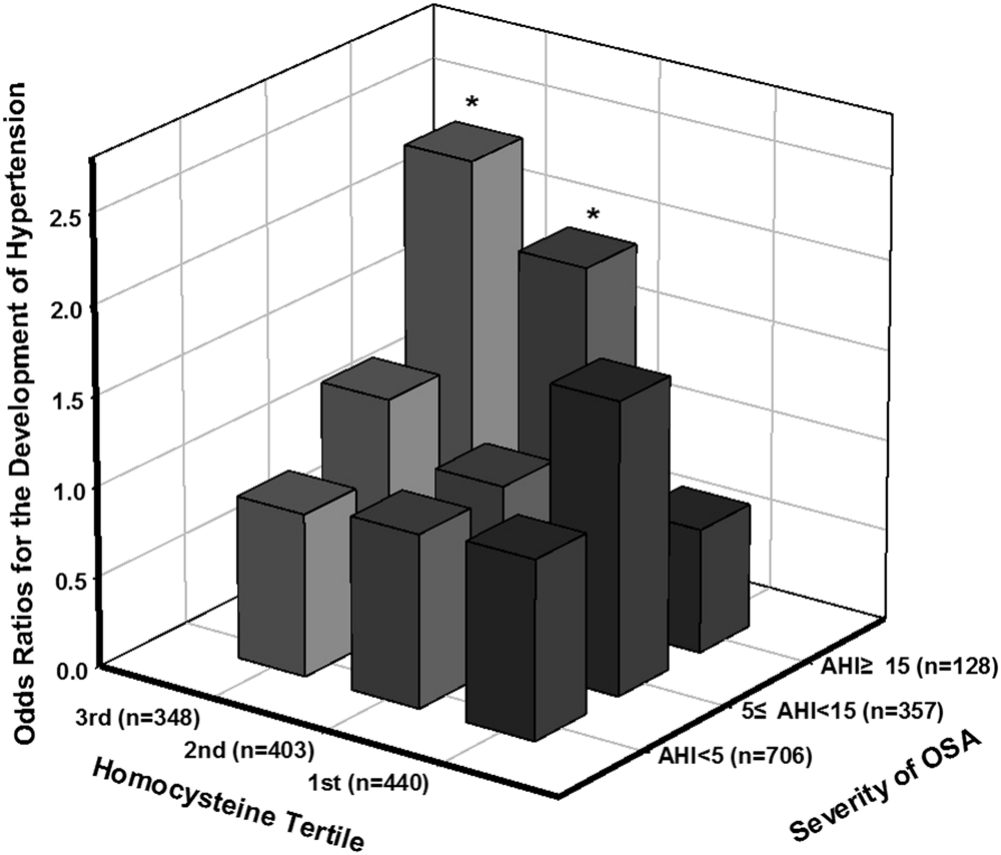
Estimated odds ratios for the development of hypertension according to the combination of the tertile homocysteine levels and severity of OSA at a 6-year follow-up. The odds ratio was estimated after adjusting for age, gender, smoking, alcohol status, BMI, DM medication, vitamin supplement intake, fasting glucose, HDL cholesterol, and triglycerides at baseline and BMI change (ΔBMI) at 6-year follow-up (n = 1191). P-value for interaction = 0.138. *p < 0.05, **p < 0.01.

Figure 1 presents the odds ratios estimated for the likelihood of hypertension according to the tertile of Hcy level and severity of OSA in participants. The multivariate analysis with adjustment for age, gender, smoking, alcohol status, BMI, DM medication, fasting glucose, HDL cholesterol, and triglyceride showed the odds ratios for hypertension among the mild OSA (5≤ AHI <15) and moderate to severe OSA group (AHI ≥15) with the highest tertile of Hcy levels (≥12.3 µmol/L) being 2.92 (95% CI, 1.84–4.62; p < 0.01) and 4.51 (95% CI, 2.55–7.97; p < 0.01), respectively compared to non-OSA participants with the lowest Hcy levels. In addition, the adjusted odds ratios for the risk of developing hypertension at the 6-year follow-up among participants classified by similar cut-off points of OSA and Hcy are shown in Fig. 2. With adjustments for age, gender, smoking, alcohol status, BMI, DM medication, vitamin supplement intake, fasting glucose, HDL cholesterol, and triglycerides at baseline as well as ΔBMI at the 6-year follow-up showed moderate to severe OSA participants with the highest tertile of Hcy levels had a 2.31-fold increased risk of developing hypertension (95% CI, 1.03–5.17; p < 0.05) compared to non-OSA participants with the lowest Hcy levels. The interaction between moderate to severe OSA and Hcy level tertile was examined for their effect on the risk of development of hypertension after adjusting for age, gender, smoking, alcohol status, BMI, DM medication, vitamin supplement intake, fasting glucose, HDL cholesterol, and triglycerides at baseline as well as ΔBMI at the 6-year follow-up. The only significant interaction between the tertile of Hcy levels and moderate to severe OSA on development of hypertension was observed (p-value for interaction = 0.035).

## Discussion

In a large population-based cohort study, we found that OSA was not only associated with increased risk of hypertension, but also independently related to developing hypertension over a 6-year follow-up period. Both the prevalence and incidence of hypertension increased in a dose-dependent manner according to OSA severity (Table 2). More importantly, prevalence and incidence of hypertension were highest in OSA participants with concurrent high Hcy levels among corresponding groups (Table 3), indicating that the combination of these conditions may play an important role in exacerbating the risk for hypertension. Even when adjusting for potential confounding factors in the logistic regression models, OSA participants with high Hcy levels had a 2.71- fold risk of hypertension at baseline and a 1.82-fold increased risk for developing hypertension at the 6-year follow-up, respectively, as compared to corresponding non-OSA participants with low Hcy levels. Interestingly, in a model that investigated the risk of hypertension as a result of OSA severity combined with Hcy tertile level, we observed that participants with both moderate to severe OSA (AHI > 15) and the highest Hcy tertile level (>12.34 µmol/L) had a 4.51-fold increased risk for hypertension and a 2.31-fold risk for developing hypertension at the 6-year follow-up compared to non-OSA participants with the lowest tertile Hcy levels after adjustment for potential confounding factors. The only significant interaction between the tertile of Hcy levels and moderate to severe OSA on developing hypertension was observed, suggesting that joint condition of moderate to severe OSA with high level of Hcy (>9.88 µmol/L) may constitute an important determinant of the development of hypertension. To our knowledge, however, this study is the first to show the coexistence effect of OSA and high Hcy levels on the development of hypertension in a prospective cohort study. Therefore, the magnitude and significance regarding the modifying effect of Hcy in OSA on the development of hypertension should be confirmed in other cohort studies.

Considering the ever-increasing body of evidence from epidemiologic and clinical studies regarding OSA, there is compelling evidence between OSA and systemic hypertension, with important implications for cardiovascular outcomes^3,6^. It has been reported that the prevalence of hypertension in patients with moderate to severe OSA ranges between 46% and 70%^6,16^, however, results from these studies were limited because of the cross-sectional design and inadequate controlling of confounders, and therefore a causal relationship between OSA and hypertension could not be established^3,6^. To date, only a few prospective studies have investigated a relationship by demonstrating a higher incidence of hypertension among subjects with OSA, however, results have been conflicting^6^. In a longitudinal study of the Wisconsin Sleep Cohort (WSC), the risk of developing hypertension in OSA participants (AHI ≥1 5) aged 30–60 years was significantly different compared to those without OSA (AHI < 5) after adjusting for age, gender, BMI, neck, and waist circumference^17^. Similarly, a large observational study included 1,889 participants who were referred to a sleep clinic and followed up for 10.1 years; this study demonstrated an increased incidence of hypertension in subjects with untreated OSA compared with treated patients^7^. In contrast to those studies, the Sleep Heart Health Study (SHHS) assessed participants in 5-year follow-up periods and revealed that subjects with an AHI greater than 30 had a higher risk of developing hypertension compared to those without OSA; however, this association was attenuated and no longer statistically significant after adjustment for BMI^8^. Another data from the longitudinal Victoria Sleep Cohort Study, a general population cohort of participants aged 30 and 70 years old, also did not observe an independent association between incidence of hypertension and OSA, suggesting that age is a strong confounder that attenuates the relationship between these two diseases. The present study found results that are similar to those of the WSC and Marin and co-workers in a Spanish population as described above^7^; we found that participants with moderate to severe OSA had a 1.74-fold increased risk of developing hypertension at the 6-year follow-up and this was statistically significant even after adjustment for age, gender, BMI, and change in BMI at follow-up. Thus, results from the present study support the hypothesis that OSA is independently associated with an increased risk for developing hypertension in the general population.

While the pathophysiologic mechanisms by which OSA leads to increased risk of cardiovascular morbidity and mortality are not clearly evident, sympathetic activation, oxidative stress due to intermittent hypoxia and reoxygenation, and low grade inflammation are proposed to play a crucial role in the pathogenesis of CVD in OSA^3–5^. Hcy is a thiol-containing intermediate amino-acid in the methionine to cysteine pathway^10^. Over the past several decades, a number of studies reported that elevation of Hcy level was related to endothelial dysfunction, arterial intimal-media wall thickening, and a pro-thrombotic state, providing a pathophysiologic explanation for the increased risk of CVD morbidity and mortality^18,19^. Considering this increasing body of evidence, OSA and homocysteine share many common pathophysiological pathways inflicting damage to the cardiovascular system. Not surprisingly, increased levels of plasma Hcy have been reported in OSA^12,13,20,21^, and such levels decreased after OSA treatment^22,23^. However, we should also emphasize that not all studies have confirmed the putative association between Hcy level and OSA^15,24^, suggesting that the causal relationship between these factors might not always be phenotypically expressed. Moreover, no study has been published asking whether co-existence of elevated Hcy levels and OSA increased the risk of hypertension and exacerbated the development of hypertension in a large prospective study. In this study, we observed that the highest prevalence and incidence of hypertension at the 6-year follow-up were among OSA participants with high Hcy levels. However, the incidence rate of hypertension among individuals with OSA and high Hcy levels relative to those with OSA and low Hcy levels might be influenced by the high rate of non-respondents among OSA subjects with high Hcy levels at the 6-year follow-up (non-respondents, n = 210; Hcy[−]/OSA[−] vs. Hcy[+]/OSA[−] vs. Hcy[−]/OSA[+] vs. Hcy[+]/OSA[+], 9.9% vs. 12.0% vs. 12.6% vs. 13.6%, p = 0.283). Moreover, since our data also showed that hsCRP levels, which recognized as an important inflammatory marker, were significantly different among groups with the presence or absence of OSA and high Hcy levels (Hcy[−]/OSA[−] vs. Hcy[+]/OSA[−] vs. Hcy[−]/OSA[+] vs. Hcy[+]/OSA[+], 0.93 ± 1.19 mg/dL vs. 1.13 ± 1.32 mg/dL vs. 1.33 ± 1.51 mg/dL vs. 1.20 ± 1.19 mg/dL, p < 0.01), we suggest that the elevation of Hcy level by activation of inflammation in OSA might potentially be accounted for. Therefore, additional studies are needed to further explore the significance of coexisting OSA and high Hcy level effects on the development of hypertension in a large prospective study.

The current study has several strengths that lend confidence to the findings. This study was a large prospective cohort study, which allowed us to establish a causal relationship. Also, we randomly selected 1825 subjects from the KoGES cohort, not from sleep clinics or from a working population, making it possible to obtain results more representative of the general population by minimizing the selection bias. The characteristics of the study population including biochemical data, BMI, Hcy levels, and diastolic BP between participants and non-participants at baseline were not significantly different (p > 0.05), but systolic BP was different (p < 0.01). Thus, our results are likely representative of the general population. Another advantage of the present study was that the evaluation of OSA was performed using a portable PSG at home, which provided a more convenient and natural sleep environment for participants to assess of OSA severity than hospital-based studies in the general population.

Although the present study included a large general population sample, prospective design, and follow-up measurements, several limitations should be acknowledged. First, the evaluation of high Hcy levels among those with OSA may not sufficiently represent the Hcy levels of participants because we did not measure Hcy levels at the 6-year follow-up. Even though a change in Hcy level for the four groups was stratified by the presence of OSA and Hcy level varied little during the 2-year follow-up period, we still believe this time span was short enough not to produce any significance change. Second, mild to moderate elevation of Hcy level in the general population may be influenced in part by a participant’s nutritional habits and exercise status^25^; these factors were not considered in the present study. Our data showed that participants with regular intake of any type of vitamin supplement had significantly lower levels of Hcy compared to those who did not take vitamin supplements. Even though the effect of vitamin supplementation on reducing the risk of CVD is still controversial^26^, several lines of research suggest that vitamin supplementation with folate, B6, and B12 has the potential to lower Hcy levels and thus cardiovascular risk^25^. In addition, genetic variances that might play an important role in the elevation of Hcy levels among those with OSA were not examined in the present study^27^. Accordingly, future longitudinal studies that focus on interactions between Hcy-related genetic variances and the development of hypertension among those with OSA should be conducted. Finally, we did not investigate the possibility of reverse causality—whether continuous positive airway pressure (CPAP) treatment and Hcy-lowering therapy for OSA could reduce the development of hypertension or any OSA-related comorbidities. Thus, further studies are needed to address this issue.

Taken together, OSA and elevated Hcy levels were associated with an increased risk of incident hypertension. Additional longitudinal study is needed to further address the significance and magnitude of the coexistence of OSA and high Hcy levels on the development of hypertension in the context of reducing CVD risk.

## Subjects and Methods

### Subjects

The Korean Genome and Epidemiology Study (KoGES), an ongoing prospective cohort study, was initiated in 2001 to examine the risk and burden of chronic disease among the general Korean population. Detailed information on the study design and aims of the KoGES has been previously reported^28–30^. In brief, from June 2001 to January 2003, a longitudinal cohort was formed, consisting of 5,015 participants who participated in a comprehensive health examination and on-site interviews at Korea University Ansan Hospital. Follow-up examinations were performed biennially with scheduled site visits. At each visit, participants signed an informed consent form and this study was approved by Korea University Ansan Hospital Human Research Committee (Protocol ID: ED0624). All methods and experiments were performed in accordance with the relevant guidelines and regulations. For the current study, data from the 4^th^ biennial examination from March 2007 to February 2009 and the 7^th^ examination from March 2013 to February 2015 (follow-up) were used. Polysomnography (PSG) was included in the study protocol in September 2009 in approximately half of KoGES participants. Although PSG will eventually be administered to the entire study population, subjects for the present study include only those with PSG data acquired between September 2009 and February 2012. After excluding participants who had missing data and those with extreme outliers of biochemical data including Hcy level, a total of 1825 individuals (959 men and 866 women) were recruited into the current study. Participants who had any known defined systemic inflammatory disease, genetic abnormality, or received any treatment for OSA were excluded. For the purpose of the present study, participants were divided into four groups based on the presence of high Hcy level among those with OSA and those without (non-OSA).

### Overnight Polysomnography

An overnight PSG was performed at each participant’s home using a portable device (Embletta® X-100; Embla Systems, San Carlos, CA, USA) as previously described^29^. Recording channels were as follows: one electroencephalography (C4-A1), one electrooculography, one chin electromyography, one modified lead II electrocardiography, one airflow from nasal airflow pressure transducer, two respiratory chest and abdominal respiratory inductance plethysmography, one pulse oximeter, and one position sensor. Obstructive apnea was defined as a clear decrease (≥90%) from baseline in the amplitude of the nasal pressure with ongoing chest and abdominal movement, and hypopneas were identified if there was a ≥30% reduction in the nasal pressure from baseline, associated with at least 4% oxygen desaturation on pulse oximetry. The duration threshold for these respiratory events was 10 sec. OSA was defined if the apnea hypopnea index (AHI) score was greater than 5 and mild OSA and moderate-to-severe OSA were defined by an AHI >5 and ≥15, respectively. Arousals were scored according to the American Academy of Sleep Medicine Scoring Manual^31,32^.

### Anthropometric and Biochemical Data

All the study participants provided personal health history, sleep habits, and lifestyle information. The daily intake of vitamin supplements was assessed by an interviewer-administered questionnaire. Daytime sleepiness was assessed with the Epworth Sleepiness Scale (ESS). Blood was drawn for biochemical analysis after overnight fasting. Plasma glucose, serum triglycerides, and HDL cholesterol were measured with an autoanalyzer (ADVIA 1650 and 1800, Siemens, Tarrytown, NY). Hcy level was measured using a chemiluminescence immunoassay assay. A “high” Hcy level was defined as >14.6 µmol for males and >11.1 µmol for females, corresponding to the 75th percentile of the whole study cohort. The population was also sub-divided into groups based on tertiles for the distribution of Hcy levels (12.34 µmol/L in the highest tertile and 9.88 µmol/L in the lowest tertile) for the subgroup analysis.

### Measurement of Blood Pressure and Definition of Hypertension

At baseline and biennial follow-up visits, blood pressure (BP) was measured by trained examiners according to a standardized protocol after a rest period of at least 5 min in the sitting position using an appropriate-sized cuff and a mercury sphygmomanometer^28^. Hypertension was defined when systolic blood pressure was 140 mmHg or higher and diastolic blood pressure was 90 mmHg or higher or taking antihypertensive medication. The prevalence of hypertension was estimated as the percentage of participants who having hypertension at baseline examination. The incidence of hypertension was defined as the percentage of participants who were newly diagnosed with hypertension during the 6-year follow-up period among those without hypertension at baseline.

### Statistical Analyses

Statistical mean difference was examined for normal variables using ANOVA, and probabilistic distribution was compared for non-normal variables by using the Kolmogorov-Smirnov test. Multivariate logistic regression was applied, adjusting for factors identified to be significant risk factors for hypertension: age, gender, smoking status, alcohol use, diabetes mellitus (DM) medication, vitamin supplement intake, fasting glucose, HDL cholesterol, triglycerides, baseline BMI, and change in BMI at the 6-year follow-up (ΔBMI). Adjusted odds ratios were estimated with 95% confidence intervals (CIs), referencing to that for the participants with neither elevated Hcy level nor OSA. Moreover, similar statistical tests were performed to assess odds ratios for hypertension according to the combination of OSA severity and the tertile levels of Hcy. Statistical significance was identified as p < 0.05. All statistical analyses were performed using SPSS (version 23.0, IBM Corp., Armonk, NY, USA).

## Electronic supplementary material

Supplementary Figure 1. Flow chart of study participants
