# Supplementary material for: Concurrent Presence of Obstructive Sleep Apnea and Elevated Homocysteine Levels Exacerbate the Development of Hypertension: A KoGES Six-year Follow-up Study
Source: Sci Rep. 2018 Feb 8;8:2665. doi: 10.1038/s41598-018-21033-5 (PMC5805705; doi:10.1038/s41598-018-21033-5)

Concurrent Presence of Obstructive Sleep Apnea and Elevated Homocysteine Levels Exacerbate the Development of Hypertension: A KoGES Six-year Follow-up Study

Jinkwan Kim, Ph.D.[1], MPH, Seung Ku Lee, Ph.D.[3], Dae Wui Yoon, Ph.D.[3], and Chol Shin, M.D.[2,3]

1. Department of Biomedical Laboratory Science, College of Health Science, Jungwon University, Geo-San, Republic of Korea

2. Department of Pulmonary Sleep and Critical Care Medicine Disorder Center, College of Medicine, Korea University, Ansan, Republic of Korea

3. Institute of Human Genomic Study, Korea University Ansan Hospital, Korea University, Ansan, Republic of Korea

Supplementary Figure 1. Flow chart of study participants

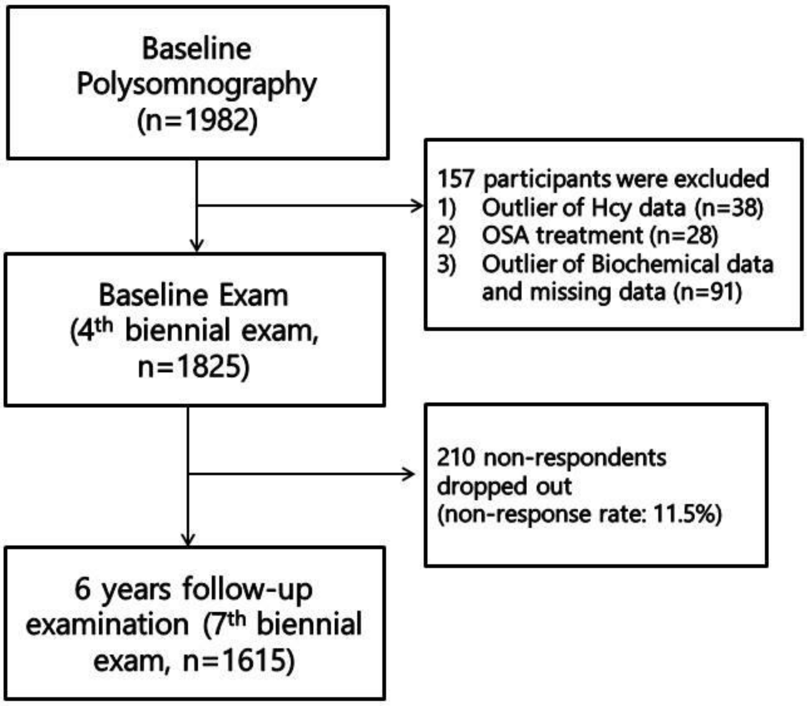

Supplement: Supplementary file 1 — Supplementary Figure 1. Flow chart of study participants [file 41598_2018_21033_MOESM1_ESM.pdf]
